# Mercury in Nelson's Sparrow Subspecies at Breeding Sites

**DOI:** 10.1371/journal.pone.0032257

**Published:** 2012-02-22

**Authors:** Virginia L. Winder, Steven D. Emslie

**Affiliations:** Department of Biology and Marine Biology, University of North Carolina Wilmington, Wilmington, North Carolina, United States of America; University of Lethbridge, Canada

## Abstract

**Background:**

Mercury is a persistent, biomagnifying contaminant that can cause negative effects on ecosystems. Marshes are often areas of relatively high mercury methylation and bioaccumulation. Nelson's Sparrows (*Ammodramus nelsoni*) use marsh habitats year-round and have been documented to exhibit tissue mercury concentrations that exceed negative effects thresholds. We sought to further characterize the potential risk of Nelson's Sparrows to mercury exposure by sampling individuals from sites within the range of each of its subspecies.

**Methodology/Principal Findings:**

From 2009 to 2011, we captured adult Nelson's Sparrows at sites within the breeding range of each subspecies (*A. n. nelsoni*: Grand Forks and Upham, North Dakota; *A. n. alterus*: Moosonee, Ontario; and *A. n. subvirgatus*: Grand Manan Island, New Brunswick) and sampled breast feathers, the first primary feather (P1), and blood for total mercury analysis. Mean blood mercury in *nelsoni* individuals captured near Grand Forks ranged from 0.84±0.37 to 1.65±1.02 SD ppm among years, between 2.0 and 4.9 times as high as concentrations at the other sites (*P*<0.01). Breast feather mercury did not vary among sites within a given sampling year (site means ranged from 0.98±0.69 to 2.71±2.93 ppm). Mean P1 mercury in *alterus* (2.96±1.84 ppm fw) was significantly lower than in any other sampled population (5.25±2.24–6.77±3.51 ppm; *P*≤0.03).

**Conclusions/Significance:**

Our study further characterized mercury in Nelson's Sparrows near Grand Forks; we documented localized and potentially harmful mercury concentrations, indicating that this area may represent a biological mercury hotspot. This finding warrants further research to determine if wildlife populations of conservation or recreational interest in this area may be experiencing negative effects due to mercury exposure. We present preliminary conclusions about the risk of each sampled population to mercury exposure.

## Introduction

Mercury biomagnifies (as methylmercury) in both aquatic and terrestrial food webs [1], [2] and can reach concentrations that result in negative effects on wildlife as well as human populations [3]–[5]. In some bird species, mercury concentrations of 2.4–40.0 ppm in feathers and 0.7–3.0 ppm in blood have been related to impaired reproduction [3], [4], [6]. Because mercury toxicity and physiology have species-specific components [3], [7], [8], and recent research indicates that we may have underestimated the effect of environmental mercury on wildlife [9], it is increasingly necessary to characterize the potential threat that mercury poses in various ecosystems. Marsh habitats are often areas of high mercury methylation and subsequent bioaccumulation because of their hydrology, acid-base status and sediment characteristics [10], [11]. As a result, omnivorous songbirds in some marsh ecosystems exhibit mercury concentrations comparable to those of piscivorous birds and terrestrial songbirds at point source contaminated sites [1], [2], [4], [8], [12].

**Figure 1:**
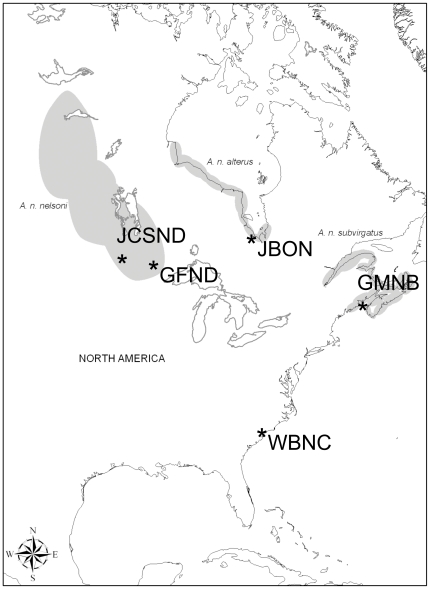
Map of breeding ranges (gray shading) for each subspecies of Nelson's Sparrow. Capture locations are noted with asterisks (*). GFND represents captures near Grand Forks, ND, USA (47°54′7.90″N, 97°17′55.31″W); GMNB represents captures from Grand Manan Island, New Brunswick (44°42′0.00″N, 66°47′60.00″W); JBON represents captures from the shore of James Bay north of Moosonee, Ontario, Canada (51°21′36.53″N, 80°25′27.79″W); JCSND represents captures at J. Clark Salyer National Wildlife Refuge near Upham, ND (48°37′7.04″N, 100°42′21.41″W); and WBNC represents pooled data from non-breeding captures of all three subspecies near Wrightsville Beach, NC (34°10′34.68″N, 77°50′22.26″W). [Source for subspecies ranges: http://bna.birds.cornell.edu/bna/species/719/articles/introduction doi:10.2173/].

Nelson's Sparrows (*Ammodramus nelsoni*) are omnivorous passerines, divided into three subspecies with geographically separate breeding ranges in freshwater wetlands and salt marshes in North America (*A. n. nelsoni*, *A. n. alterus*, *A. n. subvirgatus*; Fig. 1). All three subspecies sometimes occur together in mixed flocks in salt marshes along the coasts of the southeastern U.S. and Gulf of Mexico during the non-breeding season. Patchy wetland breeding habitats and the limited wintering range of this species have already been reduced and fragmented on the Atlantic coast of North America [13], resulting in the recognition of Nelson's Sparrow as a species of conservation concern on various watchlists [14], [15]. Mercury exposure may be an important conservation concern for Nelson's Sparrows because particularly high mercury availability has been reported in areas coinciding with degraded habitat for this species [16], [17], and mean blood and feather mercury concentrations in some populations exceed negative effects thresholds established for other species [12], [18]. Previous studies have characterized mercury exposure throughout portions of the range of Nelson's Sparrows, reporting higher than expected and geographically variable tissue mercury concentrations [12], [18]–[20]. For example, in 2009, we documented elevated blood mercury concentrations (with a mean concentration of 1.1 ppm wet weight (ww)) in breeding Nelson's Sparrows near Grand Forks, North Dakota (GFND) [12]. Blood mercury concentrations of this magnitude have been associated with a reduction in nest success of approximately 20% in Carolina Wrens (*Thryothorus ludovicianus*) [4]. Additionally, mercury bioaccumulation was higher than expected based on atmospheric deposition at this location (Mercury Deposition Network; http://nadp.sws.uiuc.edu/mdn/).

We continued to sample Nelson's Sparrows near GFND and at other sites throughout the breeding range of each Nelson's Sparrow subspecies in subsequent years with the objective of using blood, breast feathers and the first primary feather (P1) as non-destructive tools to further characterize mercury exposure in these populations. Feather mercury reflects the amount of mercury in blood at the time of feather growth, which is in turn influenced by overall body burden as muscle proteins (and accompanying mercury stores) are mobilized into blood for deposition in growing feathers [1], [21]. Based on Nelson's Sparrow molt patterns, mercury in breast feathers sampled in the breeding season should be indicative of diet (and existing mercury body burden) during the non-breeding period, and P1 mercury should integrate mercury signals from both breeding and non-breeding seasons, representing annual uptake [1], [21].

**Figure 2:**
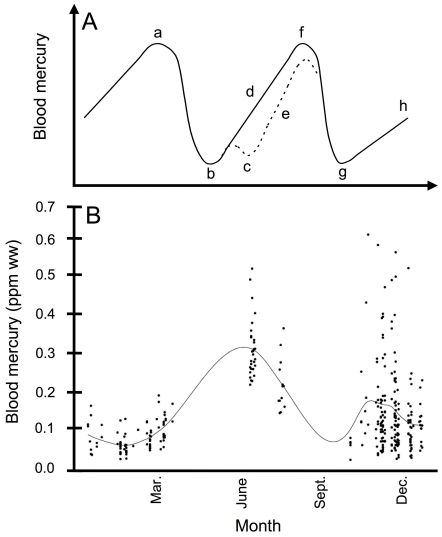
Blood mercury dynamics in a biannually molting migratory songbird. (A) Hypothetical model of changes in blood mercury with respect to physiologically dynamic events in the annual cycle of a biannually molting, migratory songbird. This simplified model assumes a constant mercury exposure year-round and is intended only to represent potential relative changes in blood mercury due to physiologically dynamic events in an individual's annual cycle. Between prebasic and prealternate molts, mercury increases during the non-breeding season (a, h); at spring prealternate molt, mercury decreases (b); females deposit mercury into eggs (c); mercury increases between prealternate and prebasic molts during the summer [d (males), e (females)], reaching a maximum before fall prebasic molt (f); following prebasic fall molt, mercury is depleted (g). (B) Blood mercury data with a smoothed best-fit line for breeding and non-breeding Nelson's Sparrow populations at JBON and WBNC, respectively (refer to Fig. 1 for capture location information).

Blood is well-suited as a biomonitoring tool for the study of mercury dynamics because it (1) allows for measurement of short-term variability in mercury uptake, (2) provides a measurement of actual contamination, and (3) reflects physiological influences such as mobilization of mercury during molt, migration, and reproduction (females only; Fig. 2A) [22]. In contrast, feather mercury concentrations represent body burden – integrated exposure over longer time frames – dampening out any short-term changes related to physiological events. Blood mercury dynamics in free-living songbirds are influenced not only by physiological events but also by varying concentrations of mercury exposure and differential prey selection across space and time. For these reasons, blood mercury data from different seasons and locations can be difficult to interpret. For example, we observed a significant decrease in blood mercury concentrations in Nelson's Sparrows in North Carolina (NC) during the non-breeding season from the time of fall arrival (October) to mid-winter (February), but we could not determine how much of this change was due to either geographic variation in mercury exposure between breeding and non-breeding sites or a seasonal diet shift [18].

The ideal methods with which to address annual blood mercury dynamics would be to track and repeatedly sample blood from a group of free-living birds at pre-determined intervals throughout the year. However, this is not yet a practical study for species with small body sizes; consequently, very little is known about the connectivity of breeding and non-breeding Nelson's Sparrow populations. We have achieved only one long-distance recapture – a male Nelson's Sparrow banded in June 2010 near Moosonee, Ontario on the shore of James Bay (JBON) that was recaptured near Wrightsville Beach, NC (WBNC) in March 2011. Because we know that these populations are connected to some degree, we believe that data from these two locations are currently the most reasonable tools with which to address questions about annual blood mercury dynamics in this species; we take this approach in the current study.

Here, we use blood and feather mercury data from breeding sites of each subspecies of Nelson's Sparrow with the objectives of determining whether (1) tissue mercury concentrations vary among breeding locations; (2) GFND and JBON tissue concentrations vary among years; and (3) GFND contamination is local or widespread. Capture of *A. n. nelsoni* at J. Clark Salyer National Wildlife Refuge (near Upham, ND; JCSND), in addition to our previous capture location (GFND), was intended to determine whether unexpectedly high 2009 GFND observations [12] were typical of the region/subspecies as a whole or specific to GFND. In addition, we use data on breeding season mercury exposure with previously published data on mercury exposure during the non-breeding season [18] to examine blood mercury dynamics and exposure risk.

## Results

**Figure 3:**
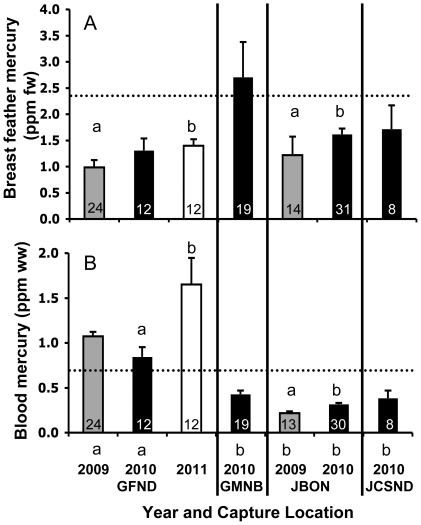
Temporal and geographic trends in breast feather and blood mercury. Mean mercury concentrations (error bars represent SD) for breeding Nelson's Sparrow (A) breast feathers and (B) blood. Refer to Fig. 1 for capture location information. Gray bars represent data from 2009, black bars 2010, and white bars 2011. Numbers within each bar represent sample size. Lowercase letters above bars represent statistical differences among years within sites (proc glm, Tukey-Kramer multiple comparisons; *P*<0.05). Lowercase letters below bars in (B) represent statistical differences in blood mercury among sites within years (proc glm, Tukey-Kramer multiple comparisons; *P*<0.05). Dashed lines represent negative effects thresholds – tissue concentrations associated with a 10% reduction in nest success in Carolina Wrens [4].

At the two locations for which we had multiple years of mercury data, GFND and JBON, breast feather and blood mercury varied between years. At GFND, 2011 breast feather mercury was significantly higher than in 2009 (*P* = 0.03; Fig. 3a), and blood mercury was higher in 2011 compared to 2010 (*P*<0.01; Fig. 3b). At JBON, 2010 breast feather mercury was significantly higher than in 2009 (*P*<0.01; Fig. 3a). Similarly, 2010 blood mercury was higher than in 2009 (*P*<0.01; Fig. 3b). P1 mercury did not vary over time at either location (Table 1).

**Table 1:** Mean first primary feather (P1) mercury concentrations (ppm fw) for Nelson's Sparrows captured at four locations (refer to Fig. 1) over three breeding seasons.

Capture location	Year	Sample Size	Mean ± SD (range)
GFND	2009	24	5.31±4.46 (0.74–14.47)
	2010	12	7.16±6.44 (1.22–21.99)
	2011	12	4.79±2.56 (2.46–11.13)
GMNB	2010	19	5.25±2.22 (1.26–9.80)
JBON	2009	14	3.27±2.36 (0.72–6.74)
	2010	30	2.66±1.26 (0.53–5.94)
JCSND	2010	8	6.77±3.51 (0.90–10.12)

Breast feather mercury did not vary among sites within a given sampling year (Fig. 3a). Sixteen percent of *subvirgatus*, 11% of *nelsoni*, and 7% of *alterus* exhibited breast feather mercury concentrations that correspond with thresholds for 10–40% reductions in nest success for Carolina Wrens (2.4–6.2 ppm fw) [4]. Additionally, 11% of *subvirgatus* exhibited breast feather concentrations that correspond with ≥50% reduction in nest success (>6.2 ppm fw) [4].

Blood mercury was significantly higher at GFND compared to JBON in 2009 and 2010 (*P*<0.01 for both pairwise comparisons) and compared to Grand Manan, New Brunswick (GMNB) and JCSND in 2010 (*P*<0.01 for both pairwise comparisons; Fig. 3b). During the three years of this study, blood mercury in 85% of individuals captured at GFND exceeded 0.7 ppm ww, and 39% exceeded 1.2 ppm ww, thresholds corresponding respectively to 10 and 20% reductions in nest success in Carolina Wrens [4]. The vast majority of birds (96%) sampled at GMNB, JBON, and JCSND had blood mercury concentrations below the lowest documented threshold for negative effects [0.7 ppm ww; 4].

P1 mercury was significantly lower at JBON compared to GFND (*P* = 0.01), GMNB (*P* = 0.01), and JCSND (*P* = 0.03; Table 1). Compared to blood and breast feather mercury, P1 concentrations indicated a higher risk to mercury exposure: 79 and 5% of *subvirgatus*, 38 and 23% of *nelsoni*, and 43 and 0% of *alterus* exhibited P1 mercury concentrations that correspond with thresholds for nest success reductions of 10–40% (3.0–9.1 ppm fw) and ≥50% (>9.1 ppm fw), respectively [4].

For GFND captures, blood mercury in 2011 and P1 mercury in 2009 were significantly higher in sparrows captured outside of the boundaries of Kellys Slough compared to those captured inside the slough (*P*<0.01 and *P* = 0.04, respectively; Table 2). All of the six GFND males captured twice in two different years were captured within the boundaries of the slough both times. In these individuals, breast feather and P1 mercury were both significantly higher at second capture compared to first capture (*P* = 0.04 for both comparisons; Table 3). Blood mercury did not change between captures in these individuals (*P* = 0.33).

**Table 2:** Mean blood and first primary feather (P1) mercury concentrations (ppm ww and fw, respectively) for Nelson's Sparrows captured near Grand Forks, ND over three breeding seasons.

Year	Slough	Sample Size	Mean Blood mercury ± SD (range)	*P*-value	Mean P1 mercury ± SD (range)	*P*-value
2009	In	19	1.02±0.17 (0.68–1.36)	0.82	4.04±3.71 (0.74–13.21)	0.04
	Out	5	1.27±0.39 (0.91–1.87)		10.15±3.94 (5.45–14.47)	
2010	In	11	0.77±0.28 (0.45–1.25)	0.12	5.81±4.64 (1.22–14.20)	0.37
	Out	1	1.67 (NA)		21.99 (NA)	
2011	In	9	1.26±0.47 (0.57–1.95)	<0.01	5.26±2.76 (2.47–11.13)	0.98
	Out	3	2.83±1.46 (1.89–4.50)		3.37±1.40 (2.46–4.98)	

Captures are divided into those that occurred within (In) the drainage basin for Kellys Slough National Wildlife Refuge and those that occurred outside of this basin (Out). Tissue mercury data for these two groups of captures were compared using generalized linear models (proc glm) with Tukey-Kramer multiple comparisons. *P*-values following each tissue mercury column are the results of these comparisons.

**Table 3:** Mean tissue mercury concentrations (ppm fw (feathers) or ww (blood)) for six Nelson's Sparrows banded and recaptured at GFND (Fig. 1) in two different breeding seasons.

Tissue	Capture	Mean tissue mercury ± SD	*P*-value
Breast feathers	1	0.87±0.22	0.04
	2	1.88±0.63	
First primary feather	1	4.56±3.93	0.04
	2	9.98±2.48	
Blood	1	0.87±0.23	0.33
	2	1.16±0.32	

Tissue mercury was compared between captures using repeated measures mixed linear models (proc mixed) incorporating capture and year as independent variables. *P*-values describe the effect of capture on tissue mercury.

Plotting blood mercury by day of year (DOY) for sparrows captured at WBNC and JBON revealed changes in mercury exposure between breeding and non-breeding seasons as well as within the non-breeding season (Fig. 2B). Blood mercury exhibited a decreasing trend from December to February before gradually increasing again in March. JBON blood mercury concentrations pushed the best-fit line above that for non-breeding concentrations, depicting a spike blood mercury during the breeding season.

## Discussion

Elevated blood mercury concentrations in Nelson's Sparrows near GFND are a sign of enhanced mercury methylation and bioaccumulation in this area. Evers et al. [23] use the term biological mercury hotspot to refer to a location where biota exhibit elevated mercury concentrations compared to surrounding areas, and these concentrations exceed wildlife health criteria. Data from the present study provide evidence that GFND exhibits both of these characteristics and as such may represent a biological mercury hotspot. In another study of mercury in birds in this region, Custer et al. [24] documented relatively low mercury concentrations in Tree Swallow (*Tachycineta bicolor*) eggs and nestlings at Lostwood National Wildlife Refuge in northwestern ND. Additionally, in 2010 (the only year for which we have data for two ND sites), mean blood mercury concentrations at GFND were 2.2 times as high as those at JCSND. These intra-regional comparisons indicate that the unexpectedly high mercury concentrations observed at GFND may be localized. In addition to being elevated compared to surrounding sites, blood mercury concentrations in Nelson's Sparrows captured at GFND exceed those documented to cause reproductive effects in other avian species [3], [4]. For example, the mean blood mercury concentration at GFND in 2011 (1.65 ppm) was near that associated with a 30% reduction in nest success in Carolina Wrens [1.7 ppm; 4], suggesting that mercury exposure at GFND is likely affecting reproduction in Nelson's Sparrows and other species inhabiting these wetlands.

Biological mercury hotspots in the northeastern United States and southeastern Canada have been attributed to elevated atmospheric mercury deposition, high landscape sensitivity and/or large reservoir fluctuations [23]. The region around GFND experiences relatively low atmospheric mercury deposition compared to biological mercury hotspots in these other regions (Mercury Deposition Network; http://nadp.sws.uiuc.edu/mdn/). However, a multitude of other factors can contribute to the creation of mercury hotspots [23]; water level fluctuation is one of these and can be an important driver of elevated mercury concentrations in wildlife [24]–[27]. During annual freeze/thaw cycles, prairie pothole wetlands alternately fill with snowmelt and seasonal rains (carrying with them runoff mercury) in spring and early summer and dry out and/or drain in late summer and fall. As soils are saturated, the anoxic environment at the soil-water interface facilitates the methylation of inorganic mercury by microbes, and the resulting organic mercury complexes with dissolved organic carbon in overlying waters, leading to bioaccumulation [25], [27]–[29]. Custer et al. [24] found that mercury concentrations were higher in seasonal wetlands compared to semi-permanent wetlands or lakes in northwestern ND. Our finding that mercury exposure was, at times, higher outside the boundaries of Kellys Slough compared to within the slough itself, also lends itself to the interpretation that fluctuations in water level are linked to increased mercury bioaccumulation because water levels and drainage patterns within the slough are more constant (with less fluctuation) than in outlying areas.

In addition to the contribution of water level fluctuations to the methylation rate of mercury, water chemistry and hydrology characteristics inherent to seasonal prairie pothole wetlands tend to increase mercury methylation rates [25]. Therefore, it seems likely that water level fluctuations and water chemistry are acting in concert to enhance mercury methylation at GFND with the end result that mercury bioaccumulation in wildlife is not proportional to its local deposition. Though a finer spatial scale study of mercury in biota would be required to unequivocally label the GFND area as a biological mercury hotspot and subsequently define its boundaries and identify its causes, our data provide preliminary evidence supporting this designation. Nelson's Sparrows have a fairly fragmented distribution within prairie wetland ecosystems, so their use as an indicator species at a finer spatial scale may not be feasible; use of a more common, widespread species may be necessary for this purpose. Regardless of the status of GFND as a biological mercury hotspot, our detection of elevated mercury bioaccumulation at this site warrants further investigation of mercury exposure in other local species of conservation concern as well as fish and game species managed for recreation and consumed by humans.

Our examination of mercury within portions of the range of each subspecies of Nelson's Sparrow also revealed geographic variation in mercury exposure, extending outside of ND. Blood mercury concentrations at GFND were not only greater than those at a neighboring site (JCSND) but were 2.0 times as high as those at GMNB in 2010 and 4.9 and 2.6 times as high as those at JBON in 2009 and 2010, respectively. *A*. *n*. *s*ubvirgatus blood mercury concentrations at GMNB (0.43±0.19 ppm ww) are remarkably similar to previously published values for this subspecies at other breeding locations (0.41 and 0.43 ppm ww) [20], [30]. These results suggest a consistent mercury exposure across the sampled portions of the breeding range of this subspecies.

Our repeated measures analysis of tissue mercury for six recaptured GFND individuals revealed that P1 and breast feather mercury concentrations more than doubled between initial capture and recapture (a period of only one year for five of the six individuals and two years for the sixth). Because we did not observe a significant interaction between year and capture (initial or recapture) in our mixed models, we cannot attribute this observed increase in feather mercury over time to temporal variation in mercury exposure. Alternatively, these data do support the explanation that elevated mercury exposure at GFND caused the annual mercury intake of these individuals to exceed their elimination capacities, thereby resulting in net annual bioaccumulation. This would result in an increasing body burden of mercury from year to year, which would be expected to be reflected in feather mercury concentrations at a subsequent capture, as we observed. Net annual bioaccumulation of mercury has been observed in Common Loons (at a rate of 8.4% year^−1^), though at a lower magnitude proportionally than we have observed here in Nelson's Sparrows, and is thought to represent an increasing risk to the negative effects of mercury exposure with age [3].

Data for each tissue type used in this study indicate that different proportions of Nelson's Sparrow subspecies populations are at risk to current concentrations of mercury exposure. The observed disparity in risk predictions among breast feathers, blood, and P1 suggests that the relationships among mercury concentrations in these tissues are different in Nelson's Sparrows and Carolina Wrens. Their respective migratory and non-migratory strategies may contribute to these differences. Differential mercury exposure between breeding and non-breeding seasons for Nelson's Sparrows may contribute to the disparity we observed between risk predictions for different tissue types in this species compared to Carolina Wrens.

Data from non-breeding NC sites [18] along with those from the present study yield a rough picture of Nelson's Sparrow mercury exposure throughout its annual cycle; both blood and breast feather mercury indicate that mercury exposure is higher on breeding compared to non-breeding sites [12], [18] (Fig. 2b). It has yet to be determined whether this difference is driven purely by geographic variation in mercury bioavailability or whether changes in diet play some part. Regardless of its cause, this non-breeding season reprieve from comparatively high mercury exposure during the breeding season may lower the annual risk of Nelson's Sparrows to mercury exposure compared to a non-migratory species, such as the Carolina Wren, that potentially faces elevated mercury exposure year-round [4].

We caution against the assumption that our results imply that the negative effects thresholds established for Carolina Wrens are meaningless in other songbird species. Conversely, we regard these thresholds as a useful starting point with which to evaluate mercury exposure when species-specific data are unavailable. The use of three tissue types to evaluate mercury exposure is advantageous in that it allows the risk to populations to be assessed with multiple measures. Until a species-specific assessment establishes negative effects thresholds for Nelson's Sparrows, it seems prudent to conservatively consider mercury data from each available tissue type to assess the risk of Nelson's Sparrow populations to mercury exposure.

As such, we make the following preliminary conclusions about the risk of each sampled Nelson's Sparrow population to mercury exposure. Blood mercury concentrations in over 95% of individuals sampled outside of GFND are below documented effects threshold for other species, suggesting that mercury exposure during the breeding season is not likely harmful in these Nelson's Sparrow populations. With respect to both feather and blood mercury, *alterus* at JBON appear to be at the lowest risk of all populations sampled in this study. Feather mercury concentrations predict that a substantial proportion of the GMNB *subvirgatus* population is at risk in spite of comparatively low blood mercury concentrations. Conservation management plans should take into account that the potential negative effects of mercury exposure to *subvirgatus* populations may be exacerbated by other environmental threats within this subspecies' breeding range such as habitat degradation and sea level rise [13], [31]. Elevated blood mercury concentrations at GFND, the fact that nearly a quarter of the sampled *nelsoni* population (including both JCSND and GFND individuals) exhibited P1 mercury concentrations in excess of those corresponding to ≥50% reduction in nest success [4], and the link between this subspecies' preferred habitat and increased mercury methylation all indicate that these populations should be regarded as at considerable risk to negative effects mercury exposure.

## Materials and Methods

### Ethics Statement

All netting, banding and sampling activities were performed under the requisite institutional, state, provincial and federal permits: University of North Carolina Wilmington Institutional Animal Care and Use Committee 2006–020 and A0910-002, NC Banding Permit 11-BB00039 and Special Research Permit (no associated permit number), ND Game and Fish Department Special Use Permit (no associated permit number), Devils Lake WMD Complex Special Use Permit 62580-09-018/10-030/11-018, Souris River Basin NWR Complex Special Use Permit 62620-2010-013, ND Game and Fish Department Scientific Collection Permit #GNF02630659/GNF02751120/GNF02918538, Environment Canada Banding Permit #10334 AC, Canadian Wildlife Service Collection Permit #CA 0252, and United States Fish and Wildlife Service Master Banding Permit 22935 and Scientific Collecting Permit MB012555-0.

### Study sites and sampling methods

We used conspecific call playback to lure Nelson's Sparrows into mist nets at four breeding sites (Fig. 1). We captured individuals of the *alterus* subspecies from 25 to 31 July 2009 (n = 14) and 26 June to 01 July 2010 (n = 30) at JBON. We captured *subvirgatus* from 02 to 05 July and 18 to 20 August 2010 (n = 11 and 8, respectively) at GMNB. We captured *nelsoni* from 20 to 24 June 2009 (n = 24), 11 to 14 June 2010 (n = 13), and 17 to 21 June 2011 (n = 17) at prairie wetland sites within Kellys Slough National Wildlife Refuge and Grand Forks County Waterfowl Management Areas and Waterfowl Production Areas (GFND) and 15 and 16 June 2010 (n = 8) at JCSND (approximately 260 km from GFND).

Nelson's Sparrows were banded with USGS aluminum bands. Blood was sampled by pricking the brachial vein with a sterile 26G1/2 needle and collecting up to 70 µl using a heparin-coated capillary tube. Capillary tubes were capped with Crito-caps® and stored in plastic vials to prevent breakage. Blood samples were initially stored on ice; after returning to the laboratory, samples were stored at −80°C until mercury analysis. The first primary feather (P1) was cut using a small pair of scissors as close to the base of the shaft as possible, and eight to ten breast feathers were plucked from each bird and stored in re-sealable plastic bags.

### Mercury analysis

To remove any externally deposited mercury, feathers were rinsed through three cycles of acetone and deionized water and allowed to dry [32]. Blood, breast feather, and P1 samples were analyzed for total mercury by thermal decomposition, catalytic conversion, gold-amalgamation, and atomic absorption spectroscopy using a Milestone® DMA-80 and a Milestone® Tri-cell DMA-80 (Shelton, CT, USA) as described in Winder and Emslie [18] using U.S. EPA Method 7473 [33]. All samples had mercury content above the minimum instrument detection limit, which ranged from 0.09 to 0.17 ng during the period of sample analysis for this study. A method blank, matrix spike (blood samples only) and standard reference material [DOLT-4 or DORM-3 (National Research Council Canada)] were run every 12–20 samples for quality assurance. Recovery of total mercury for standard reference materials ranged from 90–112%, with an average recovery of 101±1% SE. Matrix spike recovery ranged from 99–116%, averaging 107±1% SE. In the absence of adequate material for analysis of duplicate samples, matrix spikes served as a proxy for sample duplicates because recovery of mercury from both the standard reference material and sample matrix must be precise in order to achieve quality assurance results within acceptable limits.

### Statistical analyses

All statistical analyses were performed with a significance level at *P*<0.05 using SAS version 9.1. Blood, breast feather, and P1 mercury data met the assumptions for parametric statistical analyses after log_10_ transformation; therefore, log_10_ transformed concentrations were used in analyses. We present non-transformed values throughout with mercury concentrations expresses as arithmetic mean [ppm fresh weight (fw; feathers) or wet weight (ww; blood)] ± SD unless otherwise indicated. During the three years of sampling at GFND, six individuals were captured twice; to maintain independence of data for analyses addressing questions about populations, we used mercury data from only the first capture of these individuals. We did not recapture any individuals banded in previous years at any other locations.

The use of conspecific call playback resulted in the capture of nearly all males; only four of 119 captured individuals were female (two at GMNB in 2010, and one each at JBON in 2009 and 2010). Because data from Shriver et al. (2006) suggest that there is no significant difference in blood mercury concentrations between males and females of this species, we have included data for all captures in our analysis. Because GMNB sampling took place during two periods of sparrow capture, separated by approximately six weeks' time, we used a generalized linear model (glm) to test for a difference in blood mercury between these two capture periods (proc glm). Finding none (*P* = 0.75), we pooled these data into one sampling year in subsequent analyses. Because mercury in feathers is inert, and there was no evidence of initiation of the fall prebasic molt in the August captures, this was not a concern for breast feather of P1 mercury.

We used separate glms with Tukey-Kramer multiple comparisons for blood, breast feather, and P1 mercury to test whether mercury in Nelson's Sparrows varied across years at the locations for which we had multiple years of data (JBON and GFND; proc glm model: tissue mercury = year). At these locations, we observed changes in breast feather and blood mercury among years; therefore, for these two tissues, we used separate glms with Tukey-Kramer multiple comparisons to test for differences in mercury among locations within years (proc glm model: tissue mercury = location | year). P1 mercury concentrations did not vary among years at any location. Therefore, we pooled data from multiple years within locations (GFND and JBON) and used a glm with Tukey-Kramer multiple comparisons to test for differences in P1 mercury among locations (proc glm model: P1 mercury = location). For the six individuals recaptured across years at GFND, we used a mixed linear model with a repeated measures statement to test for differences in mercury concentrations between captures for each tissue (proc mixed model: tissue mercury = year | capture). Five of these six individuals were captured in successive years; the sixth was captured in 2009 and 2011.

For GFND blood and P1 mercury, preliminary examination of data indicated that individuals within the Kellys Slough boundaries (2009 n = 19, 2010 n = 11, 2011 n = 9) may have experienced lower mercury exposure than those captured outside of the slough (2009 n = 5, 2010 n = 1, 2011 n = 3); we used glms to test for this difference (proc glm model: tissue mercury = slough | year). Mercury in breast feathers sampled during the breeding season should be reflective of non-breeding mercury exposure and as such should not be influenced by breeding season exposure. Thus, we did not test whether there was a difference in breast feather mercury in individuals captured in and outside of the slough.

Finally, we examined annual blood mercury dynamics in Nelson's Sparrows using previously published data on mercury exposure during the non-breeding season at WBNC [18] and those from 2009 and 2010 at JBON (for rationale, see above). We plotted blood mercury data against DOY, fitted these data with a smoothed best-fit line using a cubic spline routine (i = sm40), and compared the resulting trends to those outlined in the hypothetical model depicting the influence of annual physiological events on blood mercury (Fig. 2a).

## References

[1] Evers D, Burgess N, Champoux L, Hoskins B, Major A (2005). Patterns and interpretation of mercury exposure in freshwater avian communities in northeastern North America.. Ecotoxicology.

[2] Cristol DA, Brasso RL, Condon AM, Fovargue RE, Friedman SL (2008). The movement of aquatic mercury through terrestrial food webs.. Science.

[3] Evers D, Savoy L, DeSorbo C, Yates D, Hanson W (2008). Adverse effects from environmental mercury loads on breeding Common Loons.. Ecotoxicology.

[4] Jackson A, Evers D, Etterson M, Condon A, Folsom S (2011). Mercury exposure affects the reproductive success of a free-living terrestrial songbird, the Carolina Wren (*Thryothorus ludovicianus*).. Auk.

[5] Clarkson T, Magos L (2006). The toxicology of mercury and its chemical compounds.. Critical Reviews in Toxicology.

[6] Brasso RL, Cristol DA (2008). Effects of mercury exposure on the reproductive success of tree swallows (*Tachycineta bicolor*).. Ecotoxicology.

[7] Bechard M, Perkins D, Kaltenecker G, Alsup S (2009). Mercury Contamination in Idaho Bald Eagles, *Haliaeetus leucocephalus*.. Bulletin of Environmental Contamination and Toxicology.

[8] Lane O, O'Brien K, Evers D, Hodgman T, Major A (2011). Mercury in breeding saltmarsh sparrows (*Ammodramus caudacutus caudacutus*).. Ecotoxicology.

[9] Evers D, Wiener J, Basu N, Bodaly R, Morrison H (2011). Mercury in the Great Lakes region: bioaccumulation, spatiotemporal patterns, ecological risks, and policy.. Ecotoxicology.

[10] Williams TP, Bubb JM, Lester JN (1994). Metal accumulation within salt marsh environments: a review.. Marine Pollution Bulletin.

[11] Marvin-DiPasquale MC, Agee JL, Bouse RM, Jaffe BE (2003). Microbial cycling of mercury in contaminated pelagic and wetland sediments of San Pablo Bay, California.. Environmental Geology.

[12] Winder V, Emslie S (2011). Mercury in breeding and wintering Nelson's Sparrows (*Ammodramus nelsoni*).. Ecotoxicology.

[13] Greenlaw JS, Woolfenden GE (2007). Wintering distributions and migration of Saltmarsh and Nelson's Sharp-tailed Sparrows.. The Wilson Journal of Ornithology.

[14] US Fish and Wildlife Service (2002). Birds of conservation concern 2002.

[15] Rich TD, Beardmore CJ, Berlanga H, Blancher PJ, Bradstreet MSW (2004). Partners in Flight North American landbird conservation plan.

[16] Evers DC, Lane OP, Savoy L, Goodale W (2004). Assessing the impacts of methylmercury on piscivorous wildlife using a wildlife criterion value based on the Common Loon, 1998–2003.

[17] Wolfe MF, Atkeson T, Bowerman WW, Burger J, Evers DC, Harris R, Krabbenhoft DP, Mason R, Murray MW, Reash R (2003). Wildlife indicators.. Ecosystem responses to mercury contamination: indicators of change.

[18] Winder VL, Emslie SD (2011). Mercury in non-breeding sparrows of North Carolina salt marshes.. Ecotoxicology.

[19] Cristol DA, Smith FM, Varian-Ramos CW, Watts BD (2011). Mercury levels of Nelson's and saltmarsh sparrows at wintering grounds in Virginia, USA.. Ecotoxicology.

[20] Shriver WG, Evers DC, Hodgman TP, MacCulloch BJ, Taylor RJ (2006). Mercury in Sharp-tailed Sparrows breeding in coastal wetlands.. Environmental Bioindicators.

[21] Bearhop S, Ruxton GD, Furness RW (2000). Dynamics of mercury in blood and feathers of great skuas.. Environmental Toxicology and Chemistry.

[22] Kahle S, Becker PH (1999). Bird blood as bioindicator for mercury in the environment.. Chemosphere.

[23] Evers DC, Han Y-J, Driscoll CT, Kamman NC, Goodale MW (2007). Biological mercury hotspots in the northeastern United States and southeastern Canada.. BioScience.

[24] Custer TW, Custer CM, Johnson KM, Hoffman DJ (2008). Mercury and other element exposure to tree swallows (*Tachycineta bicolor*) nesting on Lostwood National Wildlife Refuge, North Dakota.. Environmental Pollution.

[25] Sando SK, Krabbenhoft DP, Johnson KM, Lundgren RF, Emerson DG (2007). Mercury and methylmercury in water and bottom sediments of wetlands at Lostwood National Wildlife Refuge, North Dakota, 2003–04..

[26] Snodgrass JW, Jagoe CH, Bryan JAL, Brant HA, Burger J (2000). Effects of trophic status and wetland morphology, hydroperiod, and water chemistry on mercury concentrations in fish.. Canadian Journal of Fisheries and Aquatic Sciences.

[27] Brigham ME, Krabbenhoft DP, Olson ML, DeWild JF (2002). Methylmercury in flood-control impoundments and natural waters of northwestern Minnesota, 1997–99.. Water, Air, & Soil Pollution.

[28] Bodaly RA, Beaty KG, Hendzel LH, Majewski AR, Paterson MJ (2004). Experimenting with hydroelectric reservoirs.. Environmental Science & Technology.

[29] Driscoll CT, Han Y-J, Chen CY, Evers DC, Lambert KF (2007). Mercury contamination in forest and freshwater ecosystems in the northeastern United States.. Bio Science.

[30] Lane OP, Evers DC (2006). Methylmercury availability in New England estuaries as indicated by Saltmarsh Sharp-tailed Sparrow, 2004–2005.

[31] Bayard TS, Elphick CS (2011). Planning for sea-level Rise: Quantifying patterns of Saltmarsh Sparrow (*Ammodramus caudacutus*) nest flooding under current sea-level conditions.. Auk.

[32] Burger J (1996). Heavy metal and selenium levels in feathers of Franklin's Gulls in interior North America.. Auk.

[33] US EPA (2007). Mercury in solids and solutions by thermal decomposition, amalgamation, and atomic absorption spectrometry..

